# Endogenous Bioactive Peptides as Potential Biomarkers for Atherosclerotic Coronary Heart Disease

**DOI:** 10.3390/s120404974

**Published:** 2012-04-18

**Authors:** Takuya Watanabe, Kengo Sato, Fumiko Itoh, Kohei Wakabayashi, Masayoshi Shichiri, Tsutomu Hirano

**Affiliations:** 1. Laboratory of Cardiovascular Medicine, Tokyo University of Pharmacy and Life Sciences, 1432-1 Horinouchi, Hachioji-City, Tokyo 192-0392, Japan; E-Mails: ksato@toyaku.ac.jp (K.S.); fitoh@toyaku.ac.jp (F.I.); 2. Division of Cardiology, Showa University Fujigaoka Hospital, Yokohama, Kanagawa 227-8501, Japan; E-Mail: kwaka@live.jp; 3. Department of Endocrinology, Diabetes and Metabolism, Kitasato University School of Medicine, Sagamihara, Kanagawa 252-0374, Japan; E-Mail: shichiri@kitasato-u.ac.jp; 4. Department of Medicine, Division of Diabetes, Metabolism, and Endocrinology, Showa University School of Medicine, Tokyo 142-8555, Japan; E-Mail: hirano@med.showa-u.ac.jp

**Keywords:** acyl-coenzyme A: cholesterol acyltransferase-1, adiponectin, atherosclerosis, biomarker, coronary artery disease, glucagon-like peptide-1, heregulin-β_1_, macrophage, salusin-α

## Abstract

Cardiovascular disease is the leading cause of death worldwide, with high medical costs and rates of disability. It is therefore important to evaluate the use of cardiovascular biomarkers in the early diagnosis of coronary artery disease (CAD). We have screened a variety of recently identified bioactive peptides candidates in anticipation that they would allow detection of atherosclerotic CAD. Especially, we have focused on novel anti-atherogenic peptides as indicators and negative risk factors for CAD. *In vitro, in vivo* and clinical studies indicated that human adiponectin, heregulin-β_1_, glucagon-like peptide-1 (GLP-1), and salusin-α, peptides of 244, 71, 30, and 28 amino acids, respectively, attenuate the development and progression of atherosclerotic lesions by suppressing macrophage foam cell formation via down-regulation of acyl-coenzyme A: cholesterol acyltransferase-1. Circulating levels of these peptides in the blood are significantly decreased in patients with CAD compared to patients without CAD. Receiver operating characteristic analyses showed that salusin-α is a more useful biomarker, with better sensitivity and specificity, compared with the others for detecting CAD. Therefore, salusin-α, heregulin-β_1_, adiponectin, and/or GLP-1, alone or in various combinations, may be useful as biomarkers for atherosclerotic CAD.

## 1. Introduction

Over the past two decades, biomarkers have become increasingly utilized to improve overall patient care [1]. For example, biomarkers have had a significant impact in early detection of sub-clinical disease, diagnosis of acute or chronic syndromes, risk stratification, and in monitoring of disease and therapeutic efficacy [1]. Biomarkers are generally considered to be proteins or enzymes—measured in serum, plasma, or blood—that provide independent diagnostic and/or prognostic value by reflecting an underlying disease state [2].

Potential biomarkers have been extensively evaluated in the field of cardiovascular medicine as well as oncology [1]. Classical risk factors, such as lipids and glucose, have been well-established in coronary artery disease (CAD), while four additional markers have sufficient evidence of clinical utility to be recommended for regular clinical use: (1) cardiac troponin I and T; (2) B-type natriuretic peptides; (3) D-dimer; and (4) C-reactive protein (CRP) [1]. For example, epidemiological data demonstrated an association between high-sensitivity CRP and risk of future cardiovascular morbidity and mortality among those at high risk or with documented CAD [3].

However, only a limited number of markers have demonstrated significant diagnostic and/or therapeutic impact. Deeper insights into the pathophysiology of atherosclerosis have led to the discovery of additional novel biomarkers [1]. New vasoactive agents, inflammatory cytokines, and oxidative products that have attracted attentions have been implicated as potential biomarkers [1,2,4,5]. Previous studies have shown that high levels of pro-atherogenic vasoactive agents, such as serotonin and urotensin II, can be used as biomarkers for CAD [6,7]. Moreover, reduced circulating levels of anti-atherogenic vasoactive agents could also be used as indicators and/or negative risk factors for CAD [8,9]. In subsequent trials, we have focused on novel anti-atherogenic peptides; adiponectin, an adipocytokine [10], heregulin-β_1_ (neuregulin-1 type I), a neuron growth factor [9], glucagon-like peptide-1 (GLP-1), an incretin hormone [11], and salusin-α, a peptide recently identified by an *in silico* approach [8].

Atherosclerosis is a pathological injury-to-response process that is initiated by early inflammatory responses of vascular endothelial cells [12]. Endothelial inflammation is characterized by decreased nitric oxide production, and monocyte adhesion and infiltration into the neointima lesion, followed by oxidized low-density lipoprotein (LDL)-induced transformation of macrophages into foam cells [12]. Vascular smooth muscle cell (VSMC) and fibroblast proliferation also plays an important role in the development of atherosclerotic lesions [12]. Therefore, any potent bioactive factors modulating such pathogenetic process could possibly be clinical atherosclerotic biomarkers.

This review focuses on the protective roles of adiponectin, heregulin-β_1_, GLP-1, and salusin-α in atherosclerotic cardiovascular diseases and their emerging roles for biomarkers and therapeutic targets for CAD.

## 2. Roles in the Cardiovascular System

Human adiponectin, heregulin-β_1_, GLP-1, and salusin-α are peptides of 244, 71, 30, and 28 amino acids, respectively. Adiponectin and GLP-1 are produced predominantly by adipose tissue and the L-cells of the lower gut, respectively, and less by the cardiovascular disease [10,13]. Salusin-α and heregulin-β_1_ are both expressed in monocytes/macrophages, vascular endothelial cells, and VSMCs [9,14]. Receptors of adiponectin (AdipoR1 and AdipoR2), heregulin-β_1_ (ErbB3 and ErbB4), and GLP-1 (GLP-1R) are abundantly expressed in human monocytes and macrophages [11,15,16], endothelial cells [10,17,18], VSMCs [11,19,20], and cardiomyocytes [21–23], while salusin-α receptors have not yet been identified [8,14].

As indicated in Table 1, adiponectin, heregulin-β_1_, and GLP-1 suppress VSMC proliferation [11,20,24], show anti-inflammatory and anti-oxidant effects [18,25–29], and promote endothelial nitric oxide production [30–32]. Adiponectin, heregulin-β_1_, and GLP-1 have been shown to exhibit cardioprotective effects against ischemic injury [33–35]. GLP-1 stimulates insulin secretin from pancreatic islet β-cells and lowers blood pressure [13]. GLP-1 and adiponectin are also known to ameliorate insulin resistance, lipid metabolism, and obesity [13,36]. Salusin-α has been shown to lower blood pressure, to promote mildly VSMC and fibroblast proliferation, and to suppress cardiomyocyte apoptosis, but no effect on endothelial nitric oxide production [14,37]. Other vasoactive effects of salusin-α have not yet been clarified [8].

## 3. Anti-Atherosclerotic Effects

Interestingly, adiponectin, heregulin-β_1_, GLP-1, and salusin-α show common suppressive effects on macrophage-driven atherosclerosis. As listed in Table 2, adiponectin, heregulin-β_1_, and salusin-α suppress foam cell formation, as indicated by cholesterol ester accumulation induced by acetylated LDL in primary cultured human monocyte-derived macrophages [16,38,39]. The intracellular free cholesterol level is increased by the endocytic uptake of acetylated LDL via scavenger receptor class A (SR-A) and is decreased by efflux of free cholesterol mediated by ATP-binding cassette transporter A1 (ABCA1) [12]. As excessive accumulation of free cholesterol is toxic to cells, free cholesterol must be either removed through efflux to extracellular acceptors, such as apolipoprotein (apo) A1 and high-density lipoprotein, or esterified to cholesterol ester by the microsomal enzyme acyl-coenzyme A: cholesterol acyltransferase-1 (ACAT1) [12]. As indicated in Table 2, adiponectin, heregulin-β_1_, and salusin-α̣suppress ACAT1 expression in human monocyte-derived macrophages [16,38,39]. GLP-1 has been shown to suppress foam cell formation and ACAT1 expression in mouse macrophages [11]. Adiponectin and heregulin-β_1_, but not salusin-α, suppress SR-A expression and enhance ABCA1 expression in human monocyte-derived macrophages [16,39–41] (Table 2). Adiponectin up-regulates ABCA1 via peroxisome proliferator-activated receptor-γ (PPARγ) and liver X receptor (LXR) signaling pathways in human macrophages [42].

Further, we and other groups have documented the anti-atherosclerotic effects of adiponectin, heregulin-β_1_, GLP-1, and salusin-α by treatments of each peptide into apoE-knockout mice as an established animal model of atherosclerosis [11,16,43,44]. Treatments with adiponectin, heregulin-β_1_, GLP-1, or salusin-α significantly attenuated aortic atherosclerotic lesions accompanied with a significant decrease in macrophage infiltration [11,16,43,44]. Significant suppressions of oxidized LDL-induced foam cell formation and ACAT1 expression were documented *ex vivo* in exudate peritoneal macrophages from apoE-knockout mice infused with GLP-1 or salusin-α compared with those from vehicle-infused apoE-knockout mice [11,44]. In these experiments, GLP-1 also downregulated CD36 that contributes to the endocytic uptake of oxidized LDL into macrophages [11]. Macrophage foam cells were less observed in aortic atherosclerotic lesions from adiponectin-transgenic LDL receptor-knockout mice fed with high-fat diet [45].

## 4. Presence in Coronary Artery Atherosclerosis and Circulating Blood

Immunohistochemical analyses of human coronary arteries from patients with CAD using anti-heregulin-β_1_ or anti-salusin-α antibodies show faint staining in advanced coronary atherosclerotic lesions, suggesting decreased expression at their protein levels [16,39]. The expression of adiponectin at mRNA levels is also significantly lower in epicardial adipose tissues in CAD [46]. These findings strongly suggest that a decline in anti-atherogenic peptides may be associated with the progression of atherosclerotic lesions in human coronary arteries.

Circulating markers are more convenient for diagnosis of CAD. As specific antibodies against these peptides have been developed, their concentrations in blood samples could be quantified using radioimmunoassay and enzyme-linked immunosorbent assay (ELISA). Serum levels of total and high-molecular weight adiponectin and plasma heregulin-β_1_ levels were measured by ELISA, and their accuracy and precision were comparable among several studies [16,47–53]. The accuracy and precision of serum salusin-α and plasma GLP-1 levels measured by radioimmunoassay and ELISA were identical among each several studies [39,54–59].

To assess essential levels of peptide hormones, the factors that influence peptide production must be taken into consideration. In general, these include food intake, smoking, gender, and the presence of diabetes, hypertension or obesity. Serum salusin-α and plasma heregulin-β_1_ levels have been demonstrated to be unaffected by a number of physiological stimuli [8,9]. Since adiponectin is known to show sexual dimorphism with higher levels in women than men, von Eynatten *et al.* [48] have studied serum adiponectin levels in the limited male subjects. Because GLP-1 is temporary increased after food intake, plasma GLP-1 levels have been measured in the fasting state and/or after 75-g oral glucose tolerance test [60–62]. Similar to adiponectin measurements, we determined serum salusin-α and plasma heregulin-β_1_ levels in the fasting state [16,39].

## 5. Biomarkers for CAD

**Figure 1.**
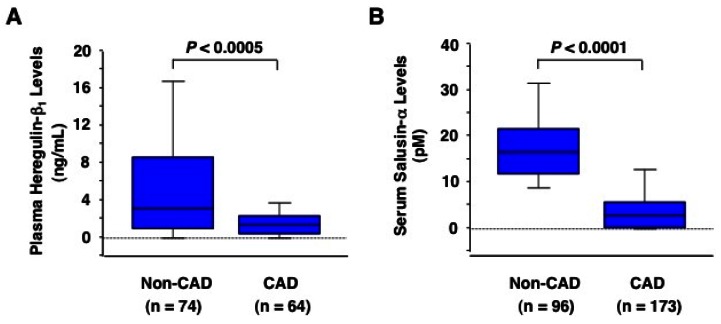
Comparisons of circulating heregulin-β_1_ and salusin-α levels between CAD and non-CAD subjects. Peripheral venous blood was sampled from patients with angiographically proven CAD, acute coronary syndrome and stable angina pectoris, and non-CAD subjects including healthy volunteers and patients with mild hypertension [16,39]. Heregulin-β_1_ and salusin-α were measured by ELISA and radioimmunoassay, respectively.

Matsubara *et al.* [60] reported that fasting plasma GLP-1 levels are significantly lower in CAD patients than in non-CAD patients (3.1 [2.4–3.6] *versus* 4.0 [3.1–5.9] pM, *P* < 0.001). Among patients without diabetes, the fasting plasma GLP-1 levels in CAD patients are significantly lower than in non-CAD patients (3.2 [2.6–3.7] *versus* 3.9 [3.0–5.2] pM, *P* < 0.001) [60]. However, Nathanson *et al.* [62] reported that impaired GLP-1 secretion after oral glucose load does not predict CAD in the presence of diabetes. El-Menyar *et al.* [63] reported that serum levels of high-molecular weight adiponectin are significantly decreased in CAD patients compared with those in non-CAD patients (1.9 ± 0.2 *versus* 3.1 ± 0.3 μg/mL, *P* = 0.003). Serum levels of high-molecular weight adiponectin were shown to be inversely correlated with angiographic severity of coronary artery lesions in patients with CAD [48]. Circulating levels of heregulin-β or salusin-α are also significantly decreased (Figure 1) and inversely correlated with angiographic severity of coronary artery lesions in patients with CAD [16,39,53].

**Figure 2.**
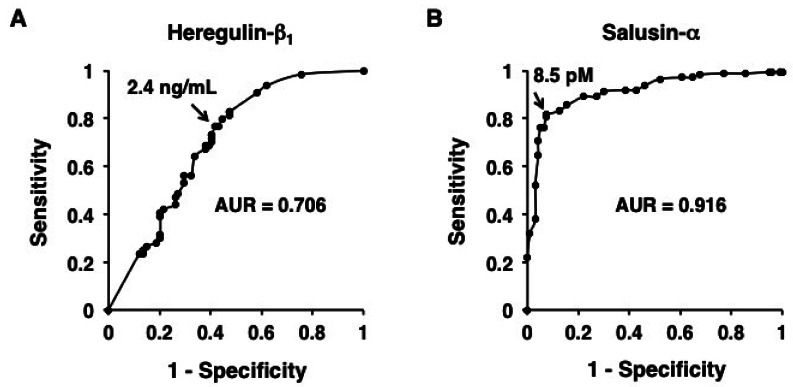
ROC curves of heregulin-β_1_ and salusin-α for detecting CAD. Based on Figure 1 data from our previous studies [16,39], ROC analyses were performed and AUC was determined in respective ROC curve. The AUC value of salusin-α is greater than that of heregulin-β_1_, indicating that salusin-α is more powerful marker for CAD than heregulin-β_1_.

Receiver operating characteristic (ROC) curves were plotted and the area under the curve (AUC) was analyzed to compare the predictive power of high-molecular weight adiponectin, heregulin-β_1_, and salusin-α. The optimal cut-off values of these peptides for detecting CAD were set at the point showing a higher true-positive rate (sensitivity) with a low false-positive rate (1-specifity) on the respective ROC curve. von Eynatten *et al.* [48] reported that the AUC value for high-molecular weight adiponectin was 0.673. The ROC curve was slightly improved by using the high-molecular weight adiponectin/total adiponectin ratio, and its AUC value became 0.718. On the basis of our previous studies [16,39], we also performed ROC analyses and calculated the AUC values in the present time. The AUC values of heregulin-β_1_ and salusin-α are 0.706 and 0.916, respectively (Figure 2). In addition, the cut-off levels were 2.4 ng/mL for heregulin-β_1_ with sensitivity and specificity of 76.6% and 58.1%, respectively, and that for salusin-α was 8.5 pM with sensitivity and specificity of 81.5% and 92.7%, respectively. In comparisons among these peptides, the AUC values for high-molecular weight adiponectin and the high-molecular weight adiponectin/total adiponectin ratio were similar to that of heregulin-β_1_ but inferior to that of salusin-α Therefore, serum salusin-α level has higher diagnostic value in detecting CAD compared with the other three peptides.

In patients with CAD, single biomarker shows somewhat high sensitivity and specificity, while the simultaneous measurement of a panel of biomarkers may increase the diagnostic accuracy [64]. Thus, these findings suggest that variously combined use of salusin-α with heregulin-β_1_, adiponectin, GLP-1, and/or other biomarkers may become the still more powerful predictor for CAD.

## 6. Cardiac Dysfunction

Circulating levels of adiponectin, heregulon-β_1_, GLP-1, and salusin-α were shown to be significantly associated with the severity of cardiac dysfunction [51,52,58,65]. Therefore, these peptides could also be potentially used as biomarkers reflecting heart failure. Elevated serum adiponectin and heregulon-β_1_ levels are associated with adverse clinical outcomes in cases of cardiac dysfunction [52,66]. Thus, further studies are required to determine their utility as biomarkers in predicting atherosclerotic CAD in the presence or absence of severe heart failure.

Recently, several studies have shown that administration of heregulin-β_1_ or GLP-1 improves cardiac dysfunction in patients with heart failure [67–71]. Intracoronary administration of adiponectin led to a reduction in myocardial infarct size and improvement of left ventricular function after ischemia/reperfusion injury in pigs [72]. However, effects of adiponectin and salusin-α on cardiac function have not yet been reported in humans. These findings provide insights into the potential use of heregulin-β_1_ or GLP-1 as an extended therapeutic window for combating refractory heart failure.

## 7. Conclusions

Adiponectin, heregulin-β_1_, GLP-1, and salusin-α could contribute to the early diagnosis and therapeutic efficacy of atherosclerosis. Decreased levels of adiponectin, heregulin-β_1_, GLP-1 and salusin-α in circulating blood and/or cardiovascular tissues are closely linked with human atherosclerosis. Thus, adiponectin, heregulin-β_1_, GLP-1, and/or salusin-α, alone or in various combinations are candidate biomarkers for predicting CAD, which may be useful for the earlier detection of atherosclerotic cardiovascular diseases.

## Figures and Tables

**Table 1.** Effects of new novel peptides on the cardiovascular system.

	Adiponectin	Heregulin-β_1_	GLP-1	Salusin-α
VSMC proliferation	↓	↓	↓	↑
eNOS	↑	↑	↑	→
Cardiomyocyte protection	+	+	+	+
Anti-inflammation	+	+	+	NE
Anti-oxidation	+	+	+	NE

GLP-1: glucagon-like peptide-1, VSMC: vascular smooth muscle cell, eNOS: endothelial nitric oxide synthase, NE: not examined. Arrows indicate either stimulation or suppression of VSMC proliferation and eNOS induction. + indicates positive effects.

**Table 2.** Effects of new novel peptides on macrophage foam cell formation.

	Adiponectin	Heregulin-β_1_	GLP-1	Salusin-α
Foam cell formation	↓	↓	↓	↓
ACAT1	↓	↓	↓	↓
SR-A	↓	↓	→	→
ABCA1	↑	↑	→	→

GLP-1: glucagon-like peptide-1, ACAT1: acyl-coenzyme A: cholesterol acyltransferase-1, SR-A: scavenger receptor class A, ABCA1: ATP-binding cassette transporter A1. Arrows indicate stimulatory, suppressive, or negative effects.
